# Prevalence of Antimicrobial Resistance Among the WHO’s AWaRe Classified Antibiotics Used to Treat Urinary Tract Infections in Diabetic Women

**DOI:** 10.3390/antibiotics13121218

**Published:** 2024-12-14

**Authors:** Ahmad Hamdan, Mohannad N. AbuHaweeleh, Leena Al-Qassem, Amira Kashkoul, Izzaldin Alremawi, Umna Hussain, Sara Khan, Menatalla M. S. ElBadway, Tawanda Chivese, Habib H. Farooqui, Susu M. Zughaier

**Affiliations:** 1 College of Medicine, QU Health, Qatar University, Doha 2713, Qatar; ah1904442@qu.edu.qa (A.H.); ma1908120@qu.edu.qa (M.N.A.); la2007753@qu.edu.qa (L.A.-Q.); ak2006143@qu.edu.qa (A.K.); ia2003723@qu.edu.qa (I.A.); uh2003540@qu.edu.qa (U.H.); sk1912664@qu.edu.qa (S.K.); ms1903450@qu.edu.qa (M.M.S.E.); 2 Department of Population Medicine, College of Medicine, QU Health, Qatar University, Doha 2713, Qatar; tchivese@qu.edu.qa (T.C.); hfarooqui@qu.edu.qa (H.H.F.)

**Keywords:** diabetes, antimicrobial resistance, antibiotics, AWaRe classification, urinary tract infection

## Abstract

**Background and Objectives**: Diabetes is linked to a higher risk of urinary tract infections (UTIs) in women, often leading to recurrent antibiotic treatments. Frequent antibiotic use for UTIs can contribute to antimicrobial resistance (AMR), a critical public health threat that increases treatment failure. This study investigated the prevalence of AMR and its associated factors among women with UTIs, comparing those with and without diabetes. **Results**: The study population had a mean age of 52 years (SD = 23) for the women without diabetes and 68 years (SD = 14) for those with diabetes. Resistance was highest for cefazolin and levofloxacin in the Access and Watch antibiotic groups, while ciprofloxacin was the most frequently prescribed antibiotic. AMR prevalence was 35.7% among the women with diabetes and 21.3% among those without. After adjustment, AMR was significantly associated with both uncomplicated diabetes (OR 1.14, 95% CI 1.08–1.21) and complicated diabetes (OR 1.54, 95% CI 1.45–1.64), as well as with higher numbers of prescribed antibiotics (OR 277.39, 95% CI 253.79–303.17). **Methods**: Using a cross-sectional cohort from the Physionet database, we analyzed data on 116,902 female participants treated for UTIs, including their antibiotic exposure, diabetes status, comorbidities, and hospital admission details. Antimicrobials were classified per the WHO’s AWaRe criteria. The primary outcome was AMR identified in urine cultures, and the association with diabetes status was evaluated using multivariable logistic regression. **Conclusions**: Our findings highlight the need for focused antimicrobial stewardship in women with diabetes to reduce the AMR rates in this vulnerable group.

## 1. Introduction

Urinary tract infections (UTIs) are one of the most prevalent infections among women in all age groups. This can be attributed to the female anatomical structure of a shorter urethra that is externally open to the vulvar vestibule close to the vaginal opening [1]. There was an estimated 404.61 million UTI cases and 236,790 deaths due to UTIs in 2019 recorded globally. The number of UTI cases has risen by 60.40% from 252.25 million in 1990 to 404.61 million in 2019 [2]. Many factors predispose individuals to UTIs, most notably diabetes mellitus (DM) [3,4].

A global rise in the prevalence of diabetes is reported, making it the most common chronic metabolic disease that leads to serious organ damage and immunosuppression [5]. The hyperglycemic state that occurs in those with diabetes reduces optimal immune system responses due to the related increased acidosis and the presence of glycosuria, providing a favorable environment for infections [6]. In women with diabetes, the incidence of UTIs is higher, and infections are often more severe and recurrent compared to non-diabetic women [7]. Although, regardless of diabetes status, uropathogenic *E. coli* is the most common pathogen responsible for UTIs [8]. This poses significant challenges in clinical management and highlights the need for improved strategies in both antibiotic stewardship and infection prevention.

Antibiotic overuse and misuse over time is the main driver of antimicrobial resistance (AMR), which has been declared one of the top ten global threats to human health [9,10]. A systematic analysis conducted in 2019 reported that AMR was the third leading cause of death, following ischemic heart disease and stroke, with an estimation of 4.95 million deaths worldwide [11]. A meta-analysis conducted in 2021, suggested that individuals with diabetes have five times higher odds of developing UTIs, and are two times more susceptible to AMR infections, when compared to individuals without diabetes [12]. Two systematic reviews and a meta-analysis from 2010 and 2014 [13,14] highlighted the effect of antibiotic consumption on the development of resistance with pooled odds ratios of 2.5 and 2.3, respectively [13,14].

The increased prevalence of AMR among uropathogens like *Escherichia coli* in diabetic women with UTIs [15] complicates management and underscores the need for careful antibiotic selection. The World Health Organization (WHO) introduced the AWaRe (Access, Watch, and Reserve) classification to guide antibiotic use and combat antimicrobial resistance (AMR) globally [16,17]. The Access group lists antibiotics with lower resistance potential, recommended as first- or second-choice treatments for common infections as they are widely accessible, affordable, and should constitute up to 60% of all antibiotic prescriptions. The Watch group lists antibiotics with higher resistance potential; thus, their use is limited to specific indications. The Reserve group lists the last-resort antibiotics used for treating confirmed or suspected infections caused by multidrug-resistant organisms and their use is highly restricted [17,18]. We examined the association between diabetes status and AMR in the female individuals with UTIs included in Physionet dataset [19,20,21] based on the WHO’s AWaRe classification of antibiotics.

## 2. Results

### 2.1. Cohort Baseline Characteristics

A total of 116,902 participants were divided into three groups, no diabetes (*n* = 94,994), uncomplicated diabetes (*n* = 13,304), and complicated diabetes (*n* = 8604). Table 1 shows the baseline characteristics of each group including race, comorbidities, prior procedures, and hospital wards where the specimens were collected. The mean age for the no diabetes group was 52 years (SD = 23) while it was 68 years (SD = 14) for both uncomplicated and complicated diabetes groups.

In this study, 72% of the participants across all the groups were of white ethnicity. The most common comorbidity among the groups was hypertension (*n* = 49,960, 43%). Most of the participants had undergone surgery prior to specimen collection (*n* = 81,363, 70%). Specimens were collected in different hospital wards; the samples collected in the outpatient setting had a higher number of participants with no diabetes and uncomplicated diabetes (56,212 and 4761, respectively) whereas the inpatient-setting samples had a higher proportion of complicated diabetes participants (*n* = 2867, 33%). The complicated diabetes group had a higher proportion of participants with comorbidities (anemia, renal disease, depression, obesity, congestive heart failure, hypertension, and others) when compared to other groups. Similarly, interventions and procedures such as a central venous catheter, surgery, mechanical ventilation, and hemodialysis were more common in the complicated diabetes groups compared to the other groups.

### 2.2. AMR Prevalence in the Reported Antibiograms (Antimicrobial Susceptibility Profiles)

**Figure 1:**
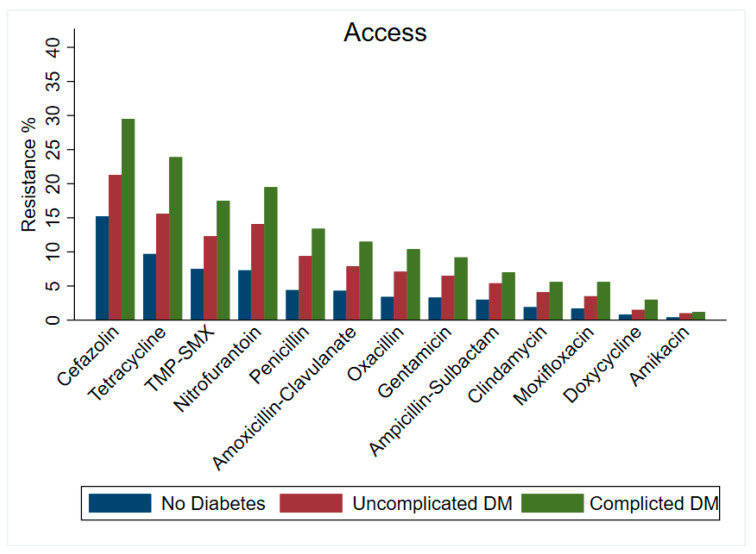
Percentage of antimicrobial resistance among antibiotics categorized under Access group within the WHO’s AWaRe classification by diabetes status. TMP-SMX: Trimethoprim-Sulfamethoxazole.

**Figure 2:**
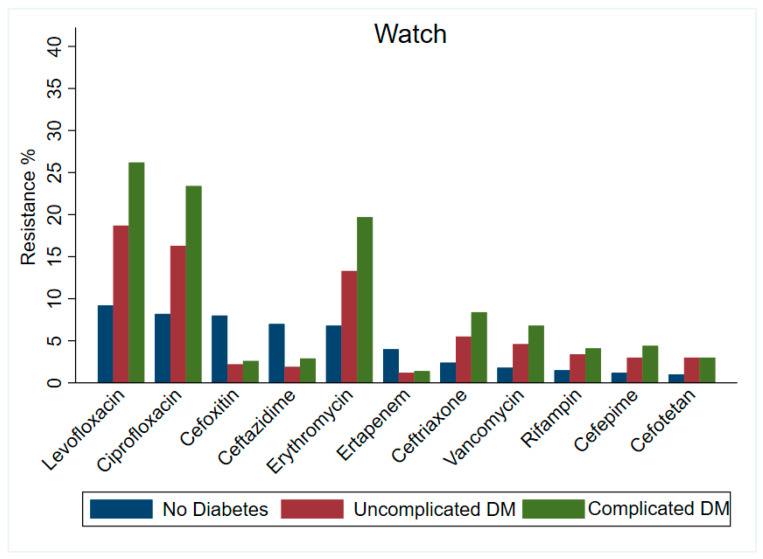
Percentage of antimicrobial resistance among antibiotics categorized under Watch group within the WHO’s AWaRe classification by diabetes status.

Our findings showed an increase in resistance rate across the groups by diabetes status with their respective WHO AWaRe classification. In the Access group (Figure 1), cefazolin showed the highest AMR among all the participants (no diabetes = 15.2%, uncomplicated diabetes = 21.3%, and complicated = diabetes 29.5%). The antibiotic with the least AMR was amikacin, in the no diabetes, uncomplicated diabetes, and complicated diabetes groups with 0.4%, 1%, and 1.2%, respectively. As for the Watch group of antibiotics (Figure 2), levofloxacin had the highest AMR, with 9.2%, 18.7%, and 26.2% among the participants with no diabetes, uncomplicated diabetes, and complicated diabetes, respectively. The antibiotic agent with the least AMR was cefotetan with 0.1% in the no diabetes group and 0.3% in both the uncomplicated and complicated diabetes groups (see Supplementary Table S1).

### 2.3. AMR Rate Across Top 5 Most Prescribed Antibiotics

To determine the effect of antibiotic usage on AMR rates, the cohort was further categorized based on number of prescribed antibiotics. The antibiotic prescription groups were categorized into one, two, three, four, and five or more antibiotics prescribed. Within each group, the top five most prescribed antibiotics were analyzed for their respective AMR rates. Table 2 shows the relationship between antibiotic usage and resistance within specific prescription groups. Across all the groups, a general trend emerged indicating an increase in AMR rate with an increase in the number of prescribed antibiotics. For example, resistance to ciprofloxacin increased sharply from 6.4% in the cases with a single antibiotic prescription to 50.9% when three antibiotics were prescribed, reaching 67.4% in those given five or more antibiotics. As ciprofloxacin resistance rose, other antibiotics with lower resistance rates were prescribed, such as cefepime and ceftriaxone from the Watch category, which showed lower resistance levels at 14.9% and 24.5%, respectively.

The pattern of antibiotic prescriptions shifted depending on how many antibiotics were given. Fluoroquinolones, particularly ciprofloxacin, were commonly prescribed, with its use ranging from 25.2% for a single antibiotic to 67.4% for five or more. As ciprofloxacin resistance increased, the antibiotics with lower resistance rates, like trimethoprim-sulfamethoxazole and nitrofurantoin, became more frequently prescribed in the initial stages. However, when more than three antibiotics were required, vancomycin, which had a lower resistance, became the most prescribed, with usage rising to 87.4%.

### 2.4. The Effect of Diabetes on AMR

Table 3 demonstrates the multivariable logistic regression model for the association between the global AMR rate (for all 26 antibiotics) and diabetes along with other covariates. The model achieved a goodness of fit represented as R^2^ = 0.36 with an AUC = 0.88 (Supplementary Figure S1). Compared to individuals without diabetes, complicated diabetes was associated with 54% higher odds of developing AMR (OR 1.54, 95% CI 1.45–1.64), while uncomplicated diabetes showed a 14% increase in the odds of developing AMR (OR 1.14, 95% CI 1.08–1.21). Similarly, an increased odds of association between the global AMR rate and the number of antibiotics prescribed was observed. For example, the odds of AMR developing were more than doubled when the prescription rate increased (ORs 13.60, 30.98, 57.25, 106.83, and 277.39 for one, two, three, four and five or more antibiotics prescribed, respectively). The hospitalized individuals demonstrated a 50% reduction in the association with AMR after adjusting for age, diabetes status, and the number of antibiotics prescribed (OR 0.48, 95% CI 0.47–0.50). Similar trends were observed in the Access group (Supplementary Table S2), the Watch group (Supplementary Table S3) and the increased number of prescriptions group (Supplementary Figure S2).

## 3. Discussion

This study’s main findings show that patients with UTIs in the uncomplicated group or complicated diabetes group consistently had higher values of antimicrobial resistance in comparison to the no diabetes group participants with UTIs. Secondly, an increased number of antibiotic prescriptions was associated with higher odds of developing AMR.

This study reports a positive relationship between diabetes status and AMR, with the odds of developing AMR being 14% and 54% higher in the uncomplicated and complicated diabetes groups, respectively. This relationship between diabetes and AMR has been previously established, although gaps remain due to its multifaceted nature particularly the effect of diabetes on increased UTI rates. In support of our data, a study conducted in Korea in 2015 [22] reported an odds of 1.36 of developing an extended spectrum beta lactamase (ESBL) *E. coli* infection from the community for individuals with diabetes as compared to those without [22]. An earlier study conducted in 2014 [23] reported similar results showing an increased odds of developing ESBL *E. coli* infections in individuals with diabetes as compared to those without, as well as in hospitalized individuals [23]. In contrast, a study published in 2018 reported a decrease in the odds of developing AMR (OR: 0.9, 95% CI: 0.4–1.8) in individuals with diabetes in a cohort of women with UTIs receiving primary care [24]. However, the results were inconclusive as the confidence intervals included the null [24].

The association between increased antibiotic prescriptions or consumption and AMR rate is well established [25,26,27]. The overuse of antibiotics imposes selective pressure on bacteria, which drives mutations in molecular drug targets leading to the evolution of AMR [28]. Additionally, Friedrich et al., reported that increased antibiotics exposure increased the AMR rates among the bacterial pathogens commonly causing healthcare-acquired infections including UTIs [29]. These multidrug resistant bacterial organisms (MDROs), commonly known as ESKAPE pathogens, carry high rates of AMR, colonize hospitalized patients’ microbiomes, and easily spread to other individuals [29]. The aforementioned facts highlight the importance of regulated and responsible antibiotic prescriptions to mitigate the risk of rising AMR rates [30,31,32].

The novelty of our study is the investigation of the relationship between complicated diabetes and antibiotics resistance, which revealed a consistent increase in resistance to all antibiotics among the participants with complicated diabetes compared to those with uncomplicated diabetes. The literature lacks studies examining the association between AMR and stratified diabetes status, while strong evidence of this relationship is reported in our study. A previous study based in Kuwait compared participants with controlled and uncontrolled glycemia [33]. However, due to its small sample size of participants, it was not possible to directly compare AMR between the controlled and uncontrolled glycemia groups [33]. The Kuwait study did find an increase in association with UTI incidence in the uncontrolled glycemia group, which is consistent with current evidence. The identified UTI risk association can be attributed to elevated glucose levels coupled with more severe immune dysregulation, which creates a favorable environment for bacterial colonization within the urinary tract. The above studies strongly advocate for a comprehensive approach to diabetes management that prioritizes glycemic control as well as emphasizing strict antibiotic prescribing practices and preventative measures to decrease the escalating threat of AMR infections in individuals with diabetes.

Our study reports high AMR rates when first-line antibiotics are used, such as nitrofurantoin and trimethoprim-sulfamethoxazole. However, our data showed that the highest rate of resistance was observed for cefazolin followed by tetracycline. In support of our results, a previous study on UTIs reported that cefazolin had the least sensitivity when used to treat common UTI-causing bacterial strains [34]. The different findings across different studies may be explained by geographical variations in the AMR trends across specific pathogens and regional antibiotic prescriptions or consumption patterns [35,36].

Another important finding of our study is that ciprofloxacin, from the Watch group classification, was consistently among the most prescribed antibiotics, regardless of the number of antibiotics prescribed. This finding may stem from ciprofloxacin’s effectiveness against a broad spectrum of the bacteria commonly causing UTIs, including the prevalent uropathogenic *Escherichia coli*. This is in agreement with the current literature, where a recent study found that bacterial resistance and the relapse rates for quinolones were significantly lower when compared with trimethoprim-sulfamethoxazole, β-lactams, and nitrofurantoin for the treatment of uncomplicated UTIs in adults [37]. In contrast, fluoroquinolones, especially ciprofloxacin, had higher resistance rates than the other antibiotics within the watch category. This might be explained by the fact that ciprofloxacin is among the most prescribed antibiotics and higher prescription rates are associated with increased odds of developing AMR.

Our results also demonstrated that vancomycin emerged as the third most prescribed antibiotic when four, five, or more antibiotics were used. This could be explained by the presence of MDROs associated with severe UTIs, which tend to progress to urosepsis, thus necessitating MRSA, and resistant *Enterococci* species coverage [38]. A recent study found that Gram-positive pathogens are common in hospitalized individuals with septic shock, thus vancomycin has been the antimicrobial drug of choice for over 60 years [39]. In our cohort, vancomycin, which is classified as a reserve antibiotic, was given only to the individuals who were prescribed five or more antibiotics, suggesting that vancomycin was withheld for use as a last resort when treating UTI cases. A similar pattern is observed for the increase in ceftriaxone usage.

The multifactorial nature of AMR in individuals with diabetes is important. Chronic inflammation, oxidative stress, and alterations in the urogenital microbiome seen in diabetic individuals contribute to the persistence and growth of resistant bacteria. Frequent hospitalization and medical device use, such as catheterization, are common in diabetic individuals and further increase their risk of developing the healthcare-associated infections caused by multidrug-resistant organisms [6]. The main contributing factors, previous antibiotic exposure and diabetes, are not mutually exclusive or independently associated with AMR. The relationship between these factors is more complex and, due to the intrinsic nature of our study, it is difficult to unravel the causal links. We propose that the immune dysregulation and suppression seen in the individuals with diabetes combined with glucosuria, serve as a favorable medium for the growth of bacteria, predisposing this group of individuals to UTIs [6,40,41]. These frequent infections necessitate frequent antibiotic prescriptions. Hence, the increased number of prescribed antibiotics led to higher odds of developing AMR. Though lacking in causality, our study uncovers the underlying effects of prescription rates that can be a driver of AMR in individuals with diabetes.

According to the European Association of Urology, an antimicrobial stewardship program should include regular staff training on the optimal use of antimicrobial agents and strict adherence to the established local, national, or international guidelines. The key elements also involve regular ward visits and consultations with infectious diseases specialists and microbiologists, along with routine audits of treatment adherence and outcomes [42]. Continuous monitoring and feedback on prescriber performance and local resistance patterns are essential to ensure effective stewardship and combat antimicrobial resistance [43]. The findings of our study recommend implementing stricter antibiotic regimens and the enhanced monitoring of antibiotic use in this at-risk group, while focusing on targeted prevention strategies to reduce infection rates, prevent the development of antimicrobial resistance, and minimize treatment failures. According to the WHO global research agenda for antimicrobial resistance, there is a need to assess and understand the patterns of antimicrobial consumption, and to promote antimicrobial stewardship efforts. Our findings help consolidate these efforts and provide quantitative evidence of how the unregulated use of antibiotics can predispose individuals to AMR [44].

It is important to acknowledge certain limitations in our study. Firstly, the research was restricted to participants exclusively from the MGH and BWH hospitals in the Northeast of the United States, which restricts the generalizability of the findings to other populations. In addition, we could not investigate the specific pathogens in our study population due to the lack of comprehensive data on UTI causative pathogens. Furthermore, the data only presented a general overview of the AMR pattern in the cohort, without specifying the individual contributions of each pathogen to the development of resistance. Moreover, the samples exhibiting intermediate resistance were labeled as resistant which could limit the interpretability of our results. Our data source (Physionet) methodology classifies the presence of any resistance as “resistant” and coded in a binary fashion with “1” referring to the presence of resistance and “0” meaning a sensitivity, regardless of the measured MIC. The substantially large size of our study population minimizes the impact of the mentioned limitations. In terms of study design, the research adopted a cross-sectional approach, which inherently limits our ability to establish causal relationships between the variables. Nevertheless, the analysis diligently accounted for various potential confounders, thereby enhancing our ability to capture the associations between the factors of interest and AMR.

Despite these limitations, the strengths of this study significantly contribute to our understanding of AMR. The large sample size and comprehensive analyses of multiple target antibiotics shed light on the current landscape of resistance patterns and can guide antimicrobial stewardship efforts. Future research could address the prescription thresholds that might help combat the AMR increase. Furthermore, prospective studies could investigate the impact of infection control measures on reducing the spread of resistant strains in addition to monitoring the change of resistance patterns as a method to invigilate the effectiveness of antimicrobial stewardship programs.

## 4. Materials and Methods

### 4.1. Study Design and Data Collection

This is a cross-sectional study using the data from Physionet [19,20,21] containing the electronic medical records of female individuals with UTIs that were treated at Massachusetts General Hospital (MGH) and Brigham and Women’s Hospital (BWH) in the USA. The data were collected between 2007 and 2016. None of the individuals in the dataset were excluded.

For all the participants, urine samples were sent to the MGH and BWH laboratories for microbiological testing. The development of AMR was determined using the minimum inhibitory concentration (MIC) and Disk Diameter (DD). These were then classified into two groups, susceptible or resistant, in accordance with the 2017 Clinical & Laboratory Standards Institute breakpoints [45]. Intermediate susceptibility was classified as resistant.

### 4.2. Exposure and Outcomes

The diagnosis of diabetes in the 6-month time window preceding specimen collection was defined as exposure. The study sample was then divided by diabetes status into three groups: no diabetes, uncomplicated diabetes, and complicated diabetes. The outcome was defined as the presence of AMR as a result of particular antibiotic use occurring within the 6 months preceding specimen collection. These three groups were compared to observe the trend of AMR development according to diabetes status with the WHO classification of antibiotics.

The antibiotics used by the participants in this study were categorized based on the World Health Organization’s AWaRe classification system [16] into Access, Watch, and Reserve categories. The dataset contained many antibiotics; however, 26 antibiotics had data on both AMR and exposure. Of these, 13 antibiotics were classified under the Access category, 11 under the Watch category, and 2 under the Reserve category. The number of antibiotics prescribed per participant was captured and categorized as follows: 0, 1, 2, 3, 4, and 5 or more, and its relationship with AMR was analyzed. The maximum number of antibiotics a participant was exposed to was 18 in the time window of the 6 months preceding specimen collection. The most frequently prescribed antibiotic in each category was captured. Global resistance was generated and defined as a test result showing resistance to any of the 26 antibiotics of interest in the time window of the 6 months preceding specimen collection. The hospitalized variable was defined as the individuals whose specimen collection was in an emergency room, an intensive care unit, or an inpatient department.

Previous diagnosis of any comorbidity within the 6 months preceding specimen collection was also captured in the dataset. The comorbidities were defined in accordance with the Elixhauser Comorbidity Index using the ICD-9 and ICD-10 codes [46]. The following comorbidities were reported in this study: anemia, metabolic syndrome, renal diseases, depression, obesity, congestive heart failure, hypertension, and other comorbidities. Any procedures done 180 days prior to, or up until the date of specimen collection were noted, including inserting a central venous catheter, surgery, mechanical ventilation, hemodialysis, and parenteral nutrition. The specific department of the hospital where the specimen was collected was also recorded, including an emergency room, intensive care unit, in-patient, or out-patient setting.

### 4.3. Statistical Analysis

The data were analyzed using STATA version SE17 (Stata Corp., College Station, TX, USA) [47]. All the categorical variables were presented as counts (*n*) and percentages (%) and group differences were compared using the Pearson’s chi-squared test. The continuous variables were presented as the means and standard deviation when normally distributed. The non-normally distributed continuous variables were displayed as medians and interquartile ranges (IQRs). An analysis of variance (ANOVA) was used to compare the continuous variables. Odds ratios (ORs) were generated using multivariable logistic regression to assess the relationship between diabetes and antimicrobial resistance with an adjustment for age. The exact *p*-values were reported. A confidence interval (CI) of 95% and the *p*-values were reported when appropriate. For the nonlinear variables, a restricted cubic spline was used to achieve linearity. The goodness of fit was assessed with an ROC graph and R^2^, and the goodness of link was assessed using a link test.

### 4.4. Ethics

The Physionet data are publicly available at https://physionet.org/content/antimicrobial-resistance-uti/1.0.0/ (accessed on 15 September 2023), containing the deidentified electronic medical records; each specimen was randomly assigned a unique ID. This study received ethics approval from the Qatar University Institutional Review Board IRB (QU-IRB 016-NR/23).

## 5. Conclusions

In a cohort of female participants treated for UTIs categorized by diabetes status, greater levels of antimicrobial resistance were observed among the participants with complicated diabetes across all the antibiotics in the WHO classified Access, Watch, and Reserve categories. Additionally, higher odds of AMR developing were associated with an increased number of antibiotics prescribed to the participants with uncomplicated and complicated diabetes when compared to the patients without diabetes.

Our study highlights the role of prescription rates and their association with AMR levels, supporting the need for enhanced antimicrobial stewardship with regard to individuals with diabetes. According to the 2023 WHO ‘Global research agenda for antimicrobial resistance in human health’, our study falls under the antibiotic use and consumption category of research priority for AMR development. Future studies are needed to investigate the effect of different pathogens on the resistance pattern in individuals with diabetes and to monitor the different regional AMR trends in this at-risk population.

## Figures and Tables

**Table 1:** Cohort baseline characteristics.

Characteristics	Categories	No Diabetes (*n* = 94,994)	Uncomplicated DM (*n* = 13,304)	Complicated DM (*n* = 8604)	*p*-Value
**Age, Mean (SD)**		52.4 (23.3)	68.3 (14.3)	68.8 (13.5)	<0.001 ^2^
**Age category, N (%)**					
	Less Than 18	4867 (5.1%)	10 (0.1%)	3 (<1%)	<0.001 ^3^
	18–39	26,507 (27.9%)	564 (4.2%)	280 (3.3%)	
	40–54	16,185 (17.0%)	1545 (11.6%)	954 (11.1%)	
	55–70	21,804 (23.0%)	4756 (35.7%)	3071 (35.7%)	
	Above 70	25,631 (27.0%)	6429 (48.3%)	4296 (49.9%)	
**Race, N (%)**					
	Other	26,677 (28.1%)	3823 (28.7%)	2635 (30.6%)	<0.001 ^3^
	White	68,317 (71.9%)	9481 (71.3%)	5969 (69.4%)	
**Comorbidity, N (%)**					
	Metabolic syndrome	6807 (7.2%)	1244 (9.4%)	586 (6.8%)	<0.001 ^3^
	Anemia	3628 (3.8%)	853 (6.4%)	1011 (11.8%)	
	Renal Disease	8771 (9.2%)	3155 (23.7%)	4158 (48.3%)	
	Depression	14,144 (14.9%)	2668 (20.1%)	2299 (26.7%)	
	Obesity	6545 (6.9%)	2456 (18.5%)	2265 (26.3%)	
	Congestive heart failure	9758 (10.3%)	3746 (28.2%)	3735 (43.4%)	
	Hypertension	32,033 (33.7%)	10,300 (77.4%)	7627 (88.6%)	
	Other Comorbidities ^1^	42,324 (44.6%)	9523 (71.6%)	6664 (77.5%)	
**Procedures, N (%)**					
	Central venous catheter	7372 (7.8%)	1830 (13.8%)	1662 (19.3%)	<0.001 ^3^
	Surgery	62,554 (65.9%)	11,223 (84.4%)	7586 (88.2%)	
	Mechanical Ventilation	6462 (6.8%)	2055 (15.4%)	1573 (18.3%)	
	Hemodialysis	732 (0.8%)	225 (1.7%)	559 (6.5%)	
	Total peripheral nutrition	1021 (1.1%)	193 (1.5%)	94 (1.1%)	
**Hospital Ward, N (%)**					
	Emergency room	17,939 (18.9%)	3608 (27.1%)	2569 (29.9%)	<0.001 ^3^
	Intensive care unit	3659 (3.9%)	891 (6.7%)	583 (6.8%)	
	In-Patient	18,036 (19.0%)	4272 (32.1%)	2867 (33.3%)	
	Out-Patient	56,212 (59.2%)	4761 (35.8%)	2776 (32.3%)	

^1^ Other comorbidities include arrhythmia, coagulopathies, hypothyroidism, liver disease, lymphoma, psychosis, peptic ulcer disease, pulmonary disease, and tumors. ^2^ *p*-value generated using Analysis of Variance (ANOVA). ^3^ *p*-value generated using Pearsons’s Chi-Squared test.

**Table 2:** Top five most prescribed antibiotics and AMR rates among participants exposed to multiple antibiotics.

Top Prescribed Antibiotics ^1^	1st Group (Use%) (Resistance%)	2nd Group (Use%) (Resistance%)	3rd Group (Use%) (Resistance%)	4th Group (Use%) (Resistance%)	5th Group (Use%) (Resistance%)
**1 (*n* = 24,234)**	Ciprofloxacin (25.2%) (6.4%)	TMP-SMX (19.0%) (7.4%)	Nitrofurantoin (13.7%) (7.1%)	Cefazolin (10.1%) (23.4%)	Levofloxacin (7.0%) (7.0%)
**2 (*n* = 12,471)**	Ciprofloxacin (42.9%) (17.3%)	TMP-SMX (29.2%) (16.6%)	Nitrofurantoin (24.8%) (15.3%)	Vancomycin (17.8%) (1.9%)	Levofloxacin (17.6%) (19.1%)
**3 (*n* = 6874)**	Ciprofloxacin (50.9%) (29.8%)	Vancomycin (39.7%) (5.4%)	TMP-SMX (32.0%) (23.3%)	Levofloxacin (31.4%) (34.0%)	Nitrofurantoin (25.6%) (24.0%)
**4 (*n* = 4422)**	Vancomycin (66.4%) (11.7%)	Ciprofloxacin (51.9%) (41.9%)	Levofloxacin (43.6%) (47.9%)	Cefepime (37.9%) (6.5%)	Ceftriaxone (34.5%) (13.3%)
**5 or more (n = 6285)**	Vancomycin (87.4%) (26.2%)	Ciprofloxacin (67.4%) (60.9%)	Levofloxacin (60.5%) (69.3%)	Cefepime (60.0%) (14.9%)	Ceftriaxone (51.7%) (24.5%)

^1^ Antibiotics exposure was investigated based on the number of prescriptions given to participants and grouped as follows: 1, 2, 3, 4, and 5 or more antibiotics. TMP-SMX: Trimethoprim-Sulfamethoxazole.

**Table 3:** Multivariable logistic regression of the effect of diabetes on AMR for all antibiotics (*n* = 116,902) ^1^.

Exposure	AMR Odds Ratio	95% Confidence Interval	*p*-Value
**Diabetes Status:**			
**No diabetes**	1		
**Uncomplicated diabetes**	1.14	1.08–1.21	<0.001
**Complicated diabetes**	1.54	1.45–1.64	<0.001
**Type of Care**			
**Outpatient**	1		
**Hospitalized**	0.48	0.47–0.50	<0.001
**Number of antibiotics prescribed**			
**No antibiotics used**	1		
**1 Antibiotic**	13.60	12.90–14.34	<0.001
**2 Antibiotics**	30.98	29.21–32.85	<0.001
**3 Antibiotics**	57.25	53.44–61.33	<0.001
**4 Antibiotics**	106.83	98.26–116.16	<0.001
**5 or more antibiotics**	277.39	253.79–303.17	<0.001

^1^ Age was adjusted for using restricted cubic splines to achieve linearity.

## Data Availability

The publicly available dataset used for this study was obtained from Physionet (https://physionet.org/) on 15 September 2023.

## Supplementary Materials

The following supporting information can be downloaded at https://www.mdpi.com/article/10.3390/antibiotics13121218/s1, Figure S1: ROC curve demonstrating the AMR prediction ability of our regression model; Table S1: Antimicrobial resistance of antibiotics classified according to WHO AWaRe classification by diabetes status; Table S2: Multivariable logistic regression of the effect of diabetes on AMR for Access antibiotics; Table S3: Multivariable logistic regression of the effect of diabetes on AMR for Watch antibiotics; Figure S2: Trends of AMR with increased number of prescriptions.
