# Supplementary material for: Prevalence of Antimicrobial Resistance Among the WHO’s AWaRe Classified Antibiotics Used to Treat Urinary Tract Infections in Diabetic Women
Source: Antibiotics (Basel). 2024 Dec 14;13(12):1218. doi: 10.3390/antibiotics13121218 (PMC11672800; doi:10.3390/antibiotics13121218)

Supplementary Material

Prevalence of Antimicrobial Resistance among the WHO's AWaRe Classified Antibiotics Used to Treat Urinary Tract Infections in Diabetic Women

Ahmad Hamdan[1*], Mohannad N. AbuHaweeleh[1*], Leena Al-Qassem[1*], Amira Kashkoul[1], Izzaldin Alremawi[1], Umna Hussain[1], Sara Khan[1], Menatalla Said[1], Tawanda Chivese[1], Habib H. Farooqui[1], Susu M Zughaier[1#]

[1]College of Medicine, QU Health, Qatar University, Doha, 2713, Qatar

[*]Authors contributed equally to this work

#Correspondence: Dr. Susu Zughaier. Department of Basic Medical Sciences, College of Medicine, Qatar University, Doha, Qatar. T: +974 4403 7859. Email: szughaier@qu.edu.qa

Authors declare no conflict of interests

Summary: This study highlights a higher prevalence of antimicrobial resistance (AMR) in women with diabetes diagnosed with UTIs, which is linked to increased antibiotic prescriptions. Findings emphasize promoting antimicrobial stewardship to reduce AMR risks, particularly within participants with diabetes.

Figure S1: ROC curve demonstrating the AMR prediction ability of our regression model.

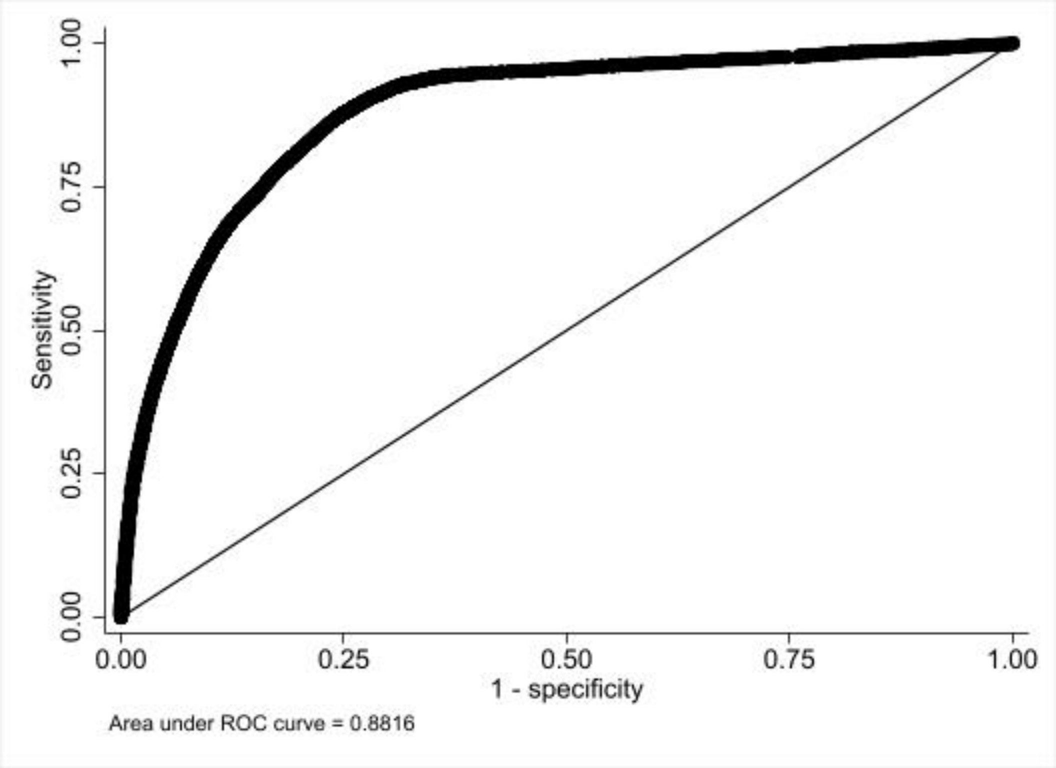

Table S1: Antimicrobial resistance of antibiotics classified according to WHO AWaRe classification by diabetes status.

Factor	No Diabetes	Uncomplicated Diabetes	Complicated Diabetes	p-value
**N**	94994	13304	8604	
**Access (n = 13)**				
**Cefazolin**	14420 (15.2%)	2830 (21.3%)	2537 (29.5%)	<0.001
**Oxacillin**	3268 (3.4%)	939 (7.1%)	893 (10.4%)	<0.001
**Tetracycline**	9219 (9.7%)	2075 (15.6%)	2058 (23.9%)	<0.001
**Penicillin**	4216 (4.4%)	1254 (9.4%)	1152 (13.4%)	<0.001
**Sulfamethoxazole-Trimethoprim**	7093 (7.5%)	1642 (12.3%)	1510 (17.5%)	<0.001
**Doxycycline**	806 (0.8%)	201 (1.5%)	261 (3.0%)	<0.001
**Nitrofurantoin**	6907 (7.3%)	1871 (14.1%)	1680 (19.5%)	<0.001
**Clindamycin**	1834 (1.9%)	551 (4.1%)	481 (5.6%)	<0.001
**Amoxicillin-Clavulanate**	4130 (4.3%)	1049 (7.9%)	988 (11.5%)	<0.001
**Gentamicin**	3089 (3.3%)	865 (6.5%)	788 (9.2%)	<0.001
**Amikacin**	334 (0.4%)	136 (1.0%)	100 (1.2%)	<0.001
**Ampicillin-Sulbactam**	2876 (3.0%)	714 (5.4%)	603 (7.0 %)	<0.001
**Moxifloxacin**	1571 (1.7%)	466 (3.5%)	479 (5.6%)	<0.001
**Watch (n = 11)**				
**Erythromycin**	6414 (6.8%)	1768 (13.3%)	1692 (19.7%)	<0.001
**Levofloxacin**	8696 (9.2%)	2484 (18.7%)	2254 (26.2%)	<0.001
**Ciprofloxacin**	7769 (8.2%)	2166 (16.3%)	2013 (23.4%)	<0.001
**Rifampin**	1397 (1.5%)	454 (3.4%)	355 (4.1%)	<0.001
**Vancomycin**	1735 (1.8%)	610 (4.6%)	587 (6.8%)	<0.001
**Ceftazidime**	692 (0.7%)	255 (1.9%)	250 (2.9%)	<0.001
**Ceftriaxone**	2301 (2.4%)	733 (5.5%)	722 (8.4%)	<0.001
**Cefepime**	1112 (1.2%)	397 (3.0%)	379 (4.4%)	<0.001
**Ertapenem**	370 (0.4%)	153 (1.2%)	120 (1.4%)	<0.001
**Cefotetan**	101 (0.1%)	36 (0.3%)	22 (0.3%)	<0.001
**Cefoxitin**	796 (0.8%)	298 (2.2%)	224 (2.6%)	<0.001
**Reserve (n = 2)**				

**Aztreonam**	1947 (2.0 %)	603 (4.5%)	581 (6.8%)	<0.001
**Linezolid**	191 (0.2%)	50 (0.4%)	57 (0.7%)	<0.001

Table S2: Multivariable logistic regression of the effect of diabetes on AMR for Access antibiotics*

AMR for Access	Odds ratio	[95% confidence interval]	p-value
**DM Status:**			
No diabetes	1		
Uncomplicated DM	1.15	1.10 - 1.22	<0.001
Complicated DM	1.52	1.43 - 1.62	<0.001
**Type of Care**			
Outpatient Care	1		
Hospitalized	0.49	0.47 - 0.51	<0.001
**Number of Antibiotics Prescribed**			
No Antibiotic	1		
1 Antibiotics	13.70	12.98-14.46	<0.001
2 Antibiotics	30.75	28.99-32.63	<0.001
3 Antibiotics	54.97	51.30-58.90	<0.001
4 Antibiotics	99.04	91.17-107.59	<0.001
5 - 18 Antibiotics	254.02	232.90-277.05	<0.001
**Constant**	0.06	0.05 - 0.07	<0.001

*Age was adjusted for using restricted cubic spline to achieve linearity.

Table S3: Multivariable logistic regression of the effect of diabetes on AMR for Watch antibiotics*

**AMR for Watch**	**Odds ratio**	**[95% confidence interval]**	**p-value**
**DM Status:**			
No diabetes	1		
Uncomplicated DM	1.23	1.16 - 1.30	<0.001
Complicated DM	1.51	1.42 - 1.61	<0.001
**Type of Care**			
Outpatient Care	1		
Hospitalized	0.94	0.90 - 0.97	0.011
**Number of Antibiotics Prescribed**			
No Antibiotic	1		
1 Antibiotics	8.05	7.43 – 8.71	<0.001
2 Antibiotics	22.70	20.97 – 24.57	<0.001
3 Antibiotics	47.70	43.87 - 51.86	<0.001
4 Antibiotics	85.11	77.67 - 93.27	<0.001
5-18 Antibiotics	221.62	202.21 - 242.89	<0.001
**Constant**	0.01	0.009 - 0.012	<0.001

*Age was adjusted for using restricted cubic spline to achieve linearity.

Figure S2: Trends of AMR with increased number of prescriptions

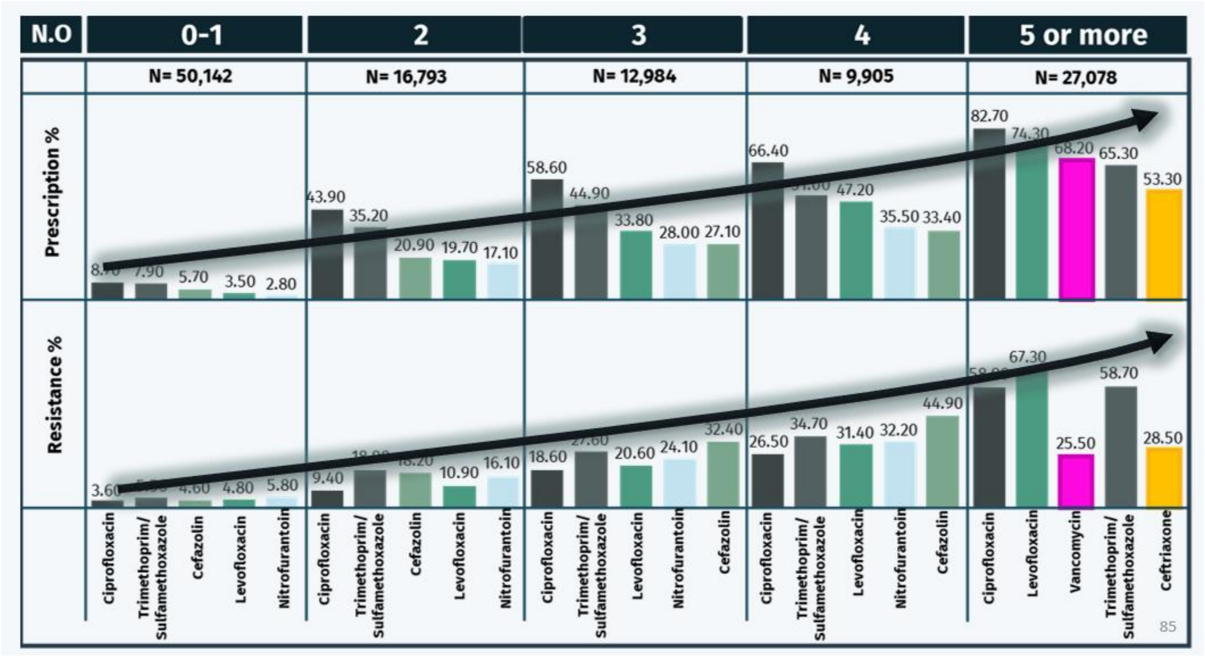

Supplement: Supplementary file 1 [file antibiotics-13-01218-s001.zip › antibiotics-3359356-supplementary.pdf]
